# Bibliographical

**Published:** 1871-12

**Authors:** 


### BIBLIOGRAPHICAL.

Among the books upon our table, which we find worthy of special notice is—

*A Treatise on Diseases of the Nervous System.* By William A. Hammond, M.D., Professor of the Diseases of the Mind and Nervous System, and of Clinical Medicine, in the Bellevue Hospital Medical College; Physician-in-Chief to the New York State Hospital for Diseases of the Nervous System, etc. With Forty-Five Illustrations.

This work is issued in fine style by D. Appleton & Co., 549 and 551 Broadway, New York, and contains seven hundred and fifty-four pages, and is a deeply interesting book, and one possessing the merit of being the record of the individual observation and experience of the author, who is regarded as the highest authority upon the subjects treated.

It embraces a consideration of the instruments and apparatus employed in the diagnosis and treatment of diseases of the nervous system, diseases of the brain, of the spinal cord, cerebro-spinal diseases, diseases of nerve-cells, and diseases of the peripheral nerves. We have been specially impressed with the views presented upon insanity, and would be pleased to know that they had reached not only every medical man, but every individual in the land.

We next call attention to

*Selected Obstetrical and Gynæcological Works of Sir James Y. Simpson, Bart., M.D., D.C.L., Late Professor of Midwifery in the University of Edinburgh:* Containing the Substance of his Lectures on Midwifery.

The book is edited by J. Watt Black, M.A., M.D., Member of the Royal College of Physicians, London; Physician Accoucheur to Charing Cross Hospital, London; and Lecturer on Midwifery and Diseases of Women and Children in the Hospital School of Medicine. This is also issued from the Press of D. Appleton & Co., 549 and 551 Broadway, New York, and contains eight hundred and fifty-two pages.

The editor of this book (Dr. Black) lived with the late Sir James Y. Simpson, as his assistant, for five years, and acquired (it is stated) during that period an intimate knowledge of his opinions, modes of practice and writings, and was requested by his representatives to compile the present volume. It contains all the more important of the author's contributions to the study of obstetrics and diseases of women, with the exception of his clinical lectures on the latter subject.

Some of the papers in the present volume are reprinted from his "Obstetric Memoirs and Contributions." His lecture notes are, however, now published in this work for the first time, and contain the substance of the practical part of his "Course of Midwifery." The simple announcement of these facts is all that can be required to arrest the attention of the profession.

We have also received, from the press of Lindsay & Blakiston, Philadelphia, the second American from the third London edition of

*Practical Therapeutics, Considered Chiefly with Reference to Articles of the Materia Medica.* By Edward John Waring, M.D., F.L.S., Member of the Royal College of Physicians, London; Fellow of the Royal College of Surgeons, England; Surgeon (Retired) in Her Majesty's Indian Army.

We have examined this work sufficiently to decide it, in our opinion, one of very great practical value, and one which meets the daily and hourly wants of the busy practitioner, who finds in short hand, not only remedies and their applications, but the authorities by whom they have been suggested or recommended, as well as the indications for the use of *certain* remedies of a *particular class*, and a rational explanation of their mode of action. It is divided into two parts, the alphabetical arrangement being adopted throughout. There is also added an index of diseases, with a list of the medicines applicable as remedies, and a full index of the medicines and preparations noticed in the work. According to the New York Medical Journal, "it is the best book of the kind ever written." It would certainly be a most valuable addition to the library of any practitioner of medicine.

